# Prominent Naturally Derived Oxidative-Stress-Targeting Drugs and Their Applications in Cancer Treatment

**DOI:** 10.3390/antiox14010049

**Published:** 2025-01-03

**Authors:** Eunsun Lee, Dongki Yang, Jeong Hee Hong

**Affiliations:** Department of Physiology, Lee Gil Ya Cancer and Diabetes Institute, College of Medicine, Gachon University, 155 Getbeolro, Yeonsu-gu, Incheon 21999, Republic of Korea; eunsunpp@gachon.ac.kr

**Keywords:** reactive oxygen species (ROS), oxidative stress, cancer therapy, angiogenesis, natural antioxidants, drug delivery systems

## Abstract

The relationship between oxidative stress and cancer has been extensively studied and highlighted, along with its role in various aspects of angiogenesis. The modulation of oxidative levels and the adaptive mechanisms of oxidative stress in cancer systems are attractive research themes for developing anti-cancer strategies. Reactive oxygen species (ROS) are involved in various pathophysiological processes and play crucial roles in DNA damage and angiogenesis. Although cancer cells have developed various adaptive defense mechanisms against oxidative stress, excessive ROS production has been proposed as an anti-cancer strategy to induce cellular apoptosis. In particular, natural-source-based antioxidants have been identified as effective against cancers, and various delivery platforms have been developed to enhance their efficacy. In this review, we highlighted the anti-cancer components (plumbagin, quercetin, resveratrol, curcumin, xanthatin, carvacrol, telmisartan, and sulforaphane) that modulate ROS levels and the recent targeting platforms used to increase the application of anti-cancer drugs and the developed delivery platforms with diverse mechanisms of action. Further, we summarized the actual doses used and the effects of these drug candidates in various cancer systems. Overall, this review provides beneficial research themes for expanding cancer-targeting fields and addressing limited applications in diverse cancer types.

## 1. Modulation of Reactive Oxygen Species Levels to Treat Cancers

Cells possess multiple antioxidant defense systems, including superoxide dismutase, thioredoxin peroxidase, catalase, glutathione peroxidase, and antioxidants including glutathione, and peroxiredoxins [1]. Adaptive responses to oxidative stress allow cancer cells to maintain redox homeostasis and promote survival and proliferation. The redox balance is modulated by the ratio of reactive oxygen species (ROS) scavengers such as glutathione, oxidized components, and oxidative-stress-associated proteins. For instance, the ratio of reduced glutathione (GSH) to oxidized glutathione (GSSG), [GSH:GSSG] is considered a marker of oxidative stress. The GSH:GSSG ratio is the highest in the serum of patients with retinoblastoma [2]. Moreover, nuclear factor erythroid 2-related factor 2 (NRF2) is associated with elements of the antioxidant response in normal and cancer cells [3]. The depleted Kirsten rat sarcoma viral oncogene homolog (K-RAS) induces a downregulated NRF2-associated mechanism and dysregulates ROS levels in lung cancer cells [4]. Based on the examples of several reported ROS scavengers, the modulation of ROS is a promising strategy for cancer therapy.

Excessive ROS disrupts redox balance, proteins, lipids, and DNA, and subsequently triggers apoptotic pathways. Increased ROS levels have been implicated in various stages of cancer progression through increased cell proliferation and motility. For instance, ROS production is associated with the proliferation and migration of prostate cancer cells [5]. Blocking ROS production using the NADPH oxidase inhibitor diphenyliodonium [5] decreases the activity of matrix metalloproteinase (MMP) 9 and mitochondrial potential and subsequently decreases cell invasive activity in prostate cancer [5]. Further, administration of the ROS hydrogen peroxide (H_2_O_2_) reduces cell viability and causes simultaneous cell cycle arrest in Calu-6 and A549 lung cancer cells [6]. These cellular reactions are accompanied by the downregulation of Bcl-2 and procaspase-3 as well as the upregulation of caspase-3 and -8 [6]. Moreover, ROS stimulation induces cell death and necrosis through cell cycle G1 phase arrest and enhances caspase activity in lung cancer cells [6]. Several studies have investigated the modulation of redox balance to treat cancer cells and identified ROS-mediated anti-cancer agents as oxidative-stress-based strategies. Additionally, the regulation of ROS production has been suggested as an effective strategy to attenuate the proliferation and invasive activity of cancers.

Naturally derived compounds are suggested to avoid drug limitations and complications, such as toxic side effects. Several naturally derived and synthetic compounds are used for ROS production to induce cytotoxic effects. In this review, we selected bioactive ROS modulators, including polyphenols and alkaloids such as plumbagin, quercetin, resveratrol, curcumin, xanthatin, carvacrol, telmisartan, and sulforaphane, to reveal their diverse mechanisms of action in various cancers, summarize their actual doses and effects, and highlight the delivery platforms used to enhance their availability.

## 2. Methodology and Approach for Systemic Review

The literature search was based on online databases, such as PubMed, Scopus, and ClinicalTrials.gov, using the selected keywords cancer, antioxidants, selected natural compounds, oxidative stress, and reactive oxygen species. Initially, we screened articles based on their titles and abstracts to assess their relevance. We specifically considered compounds that demonstrated effects and potential applications across multiple cancer types. Thus, full-text articles of potentially relevant studies involving the selected compounds of plumbagin, quercetin, resveratrol, curcumin, xanthatin, carvacrol, telmisartan, and sulforaphane were thoroughly reviewed and summarized.

## 3. ROS Mechanism-Based Natural Compounds

### 3.1. Plumbagin

**Figure 1:**
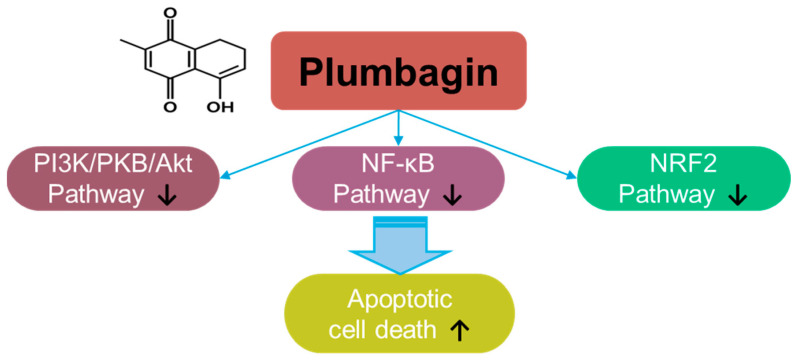
Molecular structure and mechanisms of plumbagin.

Plumbagin, a naphthoquinone compound, a hydroxy-1,4-naphthoquinone, derived from *Plumbago zeylanica* L., possesses antioxidant and anti-cancer properties [7] (Figure 1). The roles of plumbagin have been addressed in various cancers, including gastric cancer [8], colorectal cancer [9], lung cancer [10], melanoma [11], pancreatic cancer [12], hepatic cancer [13], oral squamous carcinoma [14,15], cervical carcinoma [16,17], glioma [18], and breast cancer [19,20,21]. Plumbagin treatment decreases the viability of prostate cancer cells by stimulating ROS production [22]. Further, plumbagin decreases cellular ROS metabolism-related genes, such as superoxide dismutase 2, with excessive elimination of glutathione [22]. Mechanistically, plumbagin suppresses IκB kinase α-mediated nuclear factor-κB (NF-κB) activation and subsequently inhibits the invasion of oncogene HER2-overexpressing breast cancer cells, such as the BT-474 and SK-BR-3 cell lines [23]. Plumbagin treatment also induces cell cycle arrest and apoptotic cell death by inhibiting the phosphoinositide 3-kinase (PI3K)/protein kinase B (PKB, Akt) pathway [24]. Moreover, plumbagin treatment inhibits epithelial-to-mesenchymal transition by inhibiting NRF2 [14]. More recently, combinational approaches of plumbagin with other compounds such as curcumin and cisplatin have been developed for breast cancer [25,26,27,28]. The neuroprotective effects of plumbagin in Parkinson’s disease have also been studied [29]. Therefore, the clinical use of plumbagin should be investigated in future studies.

### 3.2. Quercetin

**Figure 2:**
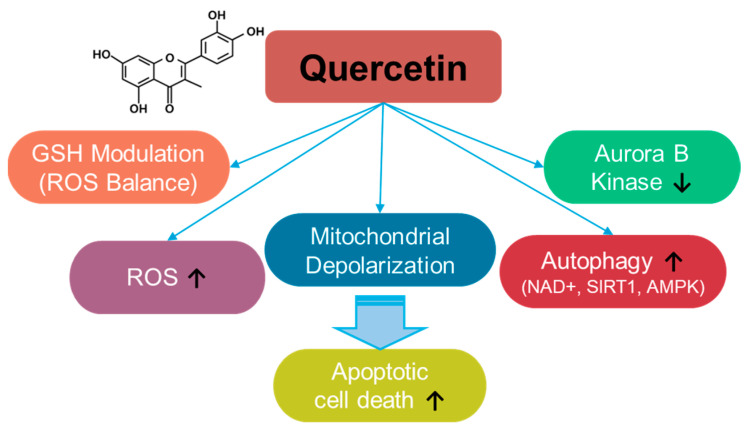
Molecular structure and mechanisms of quercetin.

Quercetin is a fruit- and vegetable-based flavonoid [30] (Figure 2) that enhances ROS levels and modulates ROS balance by altering GSH levels [31,32,33,34]. Quercetin administration increases ROS production and induces apoptosis in breast cancer cells [35]. The depletion of GSH by quercetin administration induces mitochondrial depolarization and subsequently triggers apoptosis in SW872 liposarcoma cells [34]. Further, quercetin treatment induces autophagy through enhanced nicotinamide adenine dinucleotide (NAD+)-dependent histone deacetylases, sirtuin1 (SIRT1), and AMP-activated protein kinase (AMPK) signaling in A549 and H1299 lung cancer cells [36]. In addition, tumorigenesis-associated aurora B kinase is inhibited by quercetin treatment in A549 cells and in the A549–xenograft mouse model [36,37]. The anti-cancer roles of quercetin have been elucidated in multiple cancers, including osteosarcoma, ovarian, breast, prostate, and lung cancers, and have also been reviewed in various aspects [38,39,40,41,42,43,44,45,46,47,48,49]. The effects of quercetin on various cancer systems are summarized in Table 1. However, quercetin administration presents several challenges, such as limited solubility and stability. Therefore, to improve the accessibility and therapeutic outcomes of quercetin in cancer cells, carrier-conjugated approaches such as quercetin-conjugated silver nanoparticles, liposomes, and silica nanoparticles have been suggested [50,51]. Moreover, the combination of quercetin and sulforaphane to deplete intracellular glutathione enhances anti-cancer effects on HCT116 colorectal carcinoma cell–xenograft mouse models [52]. Although the current approaches to quercetin administration have the potential to treat cancers, several challenges remain, including a lack of clinical evidence, drug resistance, and toxicity, which should be addressed carefully.

### 3.3. Resveratrol

**Figure 3:**
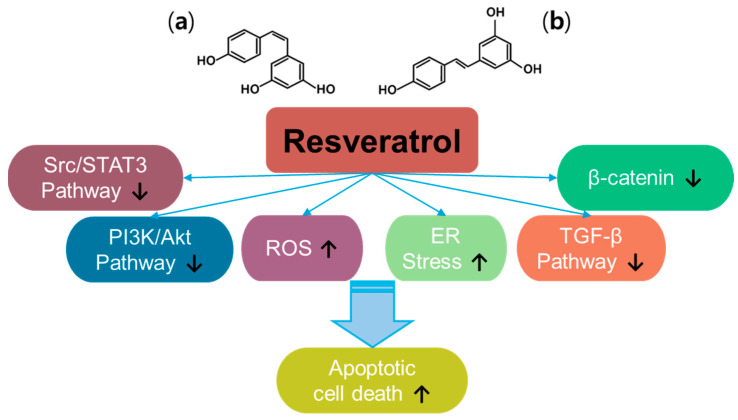
Molecular structure and mechanisms of resveratrol; (**a**) cis-resveratrol; (**b**) trans-resveratrol.

Resveratrol (trans-3, 5, 4′-trihydroxystilbene) is a plant-derived natural polyphenol product and a promising anti-cancer compound [54] (Figure 3). Resveratrol has two types of structures, the cis- and trans-forms [55]. Resveratrol has been examined in approximately 7000 articles (PubMed-based searches) that address its effects, especially against cancers, and has been reviewed extensively since its discovery [56,57,58,59]. Thus, resveratrol has been revealed to have multiple properties such as antioxidant, anti-inflammatory, and anti-cancer properties. With the different signaling pathways of resveratrol in cancers, resveratrol inhibits multiple intracellular mechanisms, such as β-catenin signaling, transforming growth factor (TGF)-β signaling, PI3K/Akt signaling, and Src/signal transducer and activator of transcription 3 (STAT3) signaling [57]. Further, resveratrol promotes ROS accumulation by regulating ROS-metabolism-associated enzymes to mediate oxidative stress and induces apoptosis in colon cancer [60]. More recently, resveratrol has been shown to induce endoplasmic reticulum (ER) stress and impair the regulation of oxidative mechanisms in A375 melanoma cells and metastatic cervical adenocarcinoma HeLa cells, respectively [61,62]. Although the beneficial effects of resveratrol have been addressed, renal toxicity in patients with myeloma and gastrointestinal side effects have been observed, indicating differences in properties depending on the cancer status or type [63,64,65,66]. Moreover, resveratrol possesses low solubility and rapid metabolism, which need to be addressed considering the pharmacological aspects of therapeutic drugs [55,57,67]. Further, resveratrol has a promising effect in increasing the sensitivity of cancers to chemo/radiotherapy [66]. Combined approaches of resveratrol and polyphenols such as epigallocatechin-3-gallate or thymoquinone (one of constituents in *Nigella sativa* black seed oil) induce synergistic anti-cancer effects in head and neck cancer models and hepatocellular carcinoma, respectively [68,69,70]. Although we did not fully highlight the anti-cancer effects of resveratrol and its delivery platforms for various cancer systems in this review, promising effects and wide applications in drug development are supported by extensive research.

### 3.4. Curcumin

**Figure 4:**
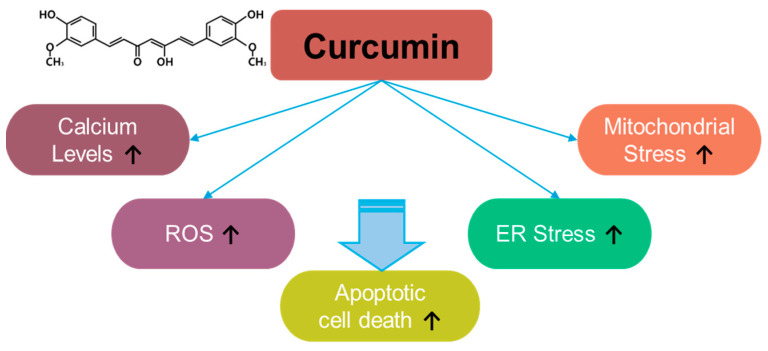
Molecular structure and mechanisms of curcumin.

Curcumin, a component of *Curcuma longa*, exerts inhibitory effects on inflammation and oxidative stress [71] (Figure 4). For instance, curcumin induces apoptosis in rat histiocytoma AK-5 cells via ROS production [71]. Moreover, in the last two decades since its discovery, multiple studies have revealed the antioxidant role of curcumin in various cancers. Although curcumin exerts a different inhibitory effect on ROS generation in methylglyoxal-exposed hepatoma G2 cells [72], it is considered a strategic component against various cancers, including, liver, oral, cervical, colon and colorectal, lung, thyroid, gastric, bladder, pancreatic, ovarian, breast, and laryngeal cancers, as well as melanoma, nasopharyngeal carcinoma, osteosarcoma, leukemia, glioma, and head and neck squamous cell carcinoma. Curcumin exerts apoptotic effects in various cancers through ROS generation, calcium increase, and increases in ER and mitochondrial stress [73]. More recently, the combination of curcumin and thymoquinone reveals anti-cancer benefits in treating breast cancer [74]. The effective doses and effects of curcumin and its combinations in various cancers are summarized in Table 2.

Although curcumin possesses effective anti-cancer properties, its pharmacological efficacy is low because of its instability and low solubility [111]. To enhance its therapeutic application, several delivery approaches, including kappa-carrageenan, nanoparticles, nanofibrous mats, halloysite nanotubes, liposomes, graphene-based nanoformulation-mediated delivery platforms, photodynamic therapy, and active metabolites, have been proposed and are summarized in Table 3.

### 3.5. Xanthatin

**Figure 5:**
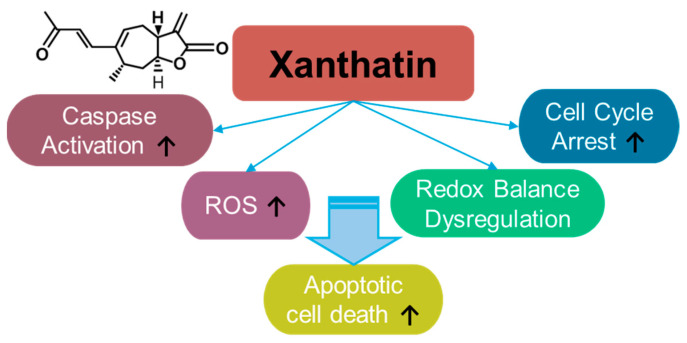
Molecular structure and mechanisms of xanthatin.

Xanthatin is a sesquiterpene lactone (Figure 5) derived from *Xanthiun strumarium* L. and exerts cytotoxic effect on cancer cells [168,169]. Since the identification of the biological properties of xanthatin, its anti-cancer effects have been demonstrated in various cancer systems such as breast cancer [170,171,172,173], gastric carcinoma [174], lung cancer [175,176,177,178], melanoma [179], colon cancer [180,181,182,183], hepatocellular carcinoma [184,185,186], pancreatic cancer [187], and glioma [188,189]. Xanthatin induces cellular apoptosis through mitochondrial ROS accumulation and the dysregulation of redox balance [178]. Moreover, xanthatin induces caspase activation and cell cycle arrest [174]. The effective doses and effects of xanthatin on various cancers are summarized in Table 4. In addition to its effects on cancer, xanthatin derivatives have been considered as potential antifungal agents [190,191] and anti-asthmatic drugs [192]. More recently, the extracted fraction of *Xanthium mongolicum* showed anti-rheumatic activity by attenuating macrophage polarity [193].

### 3.6. Carvacrol

**Figure 6:**
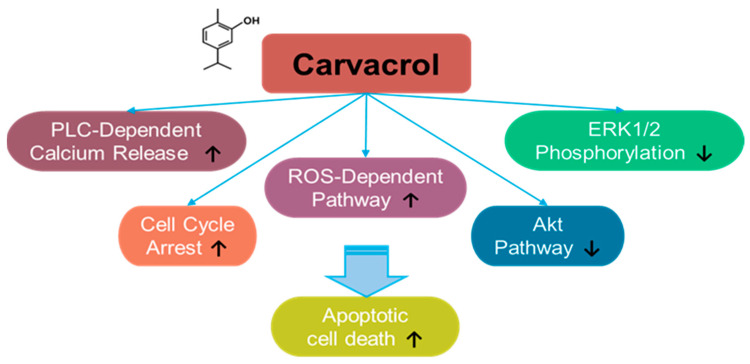
Molecular structure and mechanisms of carvacrol.

Carvacrol is a monoterpene phenol component derived from natural aromatic or herbal plants, such as *Carum copticum* and *Origanum vulgare* [194,195,196,197,198] (Figure 6). Moreover, carvacrol is a major component of oregano essential oil, which is commonly used as a dietary supplement [194]. Carvacrol has been shown to exert a promising effect in cancer treatment, in addition to its anti-inflammatory effect. For instance, the effect of carvacrol on the cancer was addressed for the first time in hepatocarcinoma cells [195]. Carvacrol treatment inhibits ERK1/2 phosphorylation and induces apoptosis in HepG2 hepatic carcinoma cells [195]. The apoptotic effects of carvacrol have also been demonstrated in DBTRG-05MG and U87 human glioblastoma cells, OC2 oral cancer cells, and HOS and U2OS osteosarcoma cells through an increase in intracellular calcium and ROS generation [199,200,201,202]. Carvacrol-induced calcium increase is mediated by phospholipase C (PLC)-dependent calcium release from calcium stores and extracellular calcium in OC2 cells [200]. Further, the proliferation and migration of HCT 116 and LoVo colon cancer cells are inhibited by carvacrol treatment through cell cycle arrest and mitochondrial apoptosis [203].

The effect of carvacrol on plasma membrane calcium channels has been investigated in several cellular systems. Carvacrol treatment blocks the current of transient receptor potential melastatin 7 (TRPM7) in TRPM7-overexpressed HEK293 cells and the TRPM7-like current in U87 glioblastoma cells [202]. U87 glioblastoma cells overexpress TRPM7 compared with those in normal astrocytes [202]. Moreover, carvacrol treatment inhibits the proliferation and migration of glioblastoma cells by inhibiting the TRPM7-mediated mitogen-activated protein kinase (MAPK) and PI3K/Akt signaling pathways [202]. A similar approach has been used for prostate cancer. Carvacrol treatment inhibits TRPM7-like current and subsequently blocks the proliferation, migration, and invasion of PC-3 and DU145 prostate cancer cells [204]. In addition, carvacrol-mediated apoptosis was mediated by PLC-dependent calcium release, cell cycle arrest, and ROS-dependent pathways through inhibition of ERK1/2 and Akt pathways in prostate cancer PC-3 and DU145 cells [205,206,207]. In Tca-8113, SCC-25, and OC2 oral squamous cell carcinoma cells, carvacrol treatment inhibits migration and invasion by inhibiting cell cycle regulation and MMP signaling [200,208]. Recently, the anti-proliferative effect of carvacrol treatment on breast cancer cells has been demonstrated. Carvacrol treatment induces breast cancer cell apoptosis by modulating the mitochondrial apoptotic genes Bax and Bcl-2 in MCF-7 cells [209].

To develop the clinical use of carvacrol against partial solubility and pharmacological stability, recent studies have suggested conjugation- or nano-based techniques. For instance, carvacrol has been conjugated with several components, such as a copper–Schiff base complex in A549 lung cancer cells [210], triphenylantimony (V) complex in MCF-7 breast cancer cells [211], hydroxypropyl-β-cyclodextrin complex in HCT116 colorectal carcinoma cells [212], and selenium/chitosan/polyethylene glycol complex in U266 myeloma cells [213]. Moreover, carvacrol was loaded onto chitosan-based nanoparticles to improve drug efficacy in breast cancer MCF-7 and HeLa cells [214].

These findings indicate that carvacrol treatment mechanistically induces apoptotic signals through caspase activation and ROS-mediated mitochondrial dysregulation. A controversial study on the anti-cancer effects of carvacrol on cervical cancer indicated that co-treatment with carvacrol and cisplatin increased HeLa cervical cancer cell viability compared to that of HeLa cells treated with cisplatin alone [215]. Although we included this controversial study in this review, carvacrol-mediated cisplatin resistance needs to be verified in future studies.

The effective doses and effects of carvacrol on various cancers are summarized in Table 5. In this review, although we highlight the anti-cancer applications of carvacrol, its potential for application in non-cancer systems is also notable. Carvacrol possesses multiple targets. In addition to being a TRPM7 antagonist [202], carvacrol is also considered a TRP ankyrin 1 (TRPA1) agonist [216]. TRPA1 signaling has been studied in skin diseases such as psoriasis [217]. Moreover, the therapeutic effects of carvacrol on skin differentiation have been studied in TRP vanilloid 3 (TRPV3)-knockout mouse skin [218]. Therefore, detailed investigations on the effects of carvacrol on channelopathy-associated skin diseases, such as psoriasis, are warranted in future.

### 3.7. Telmisartan

**Figure 7:**
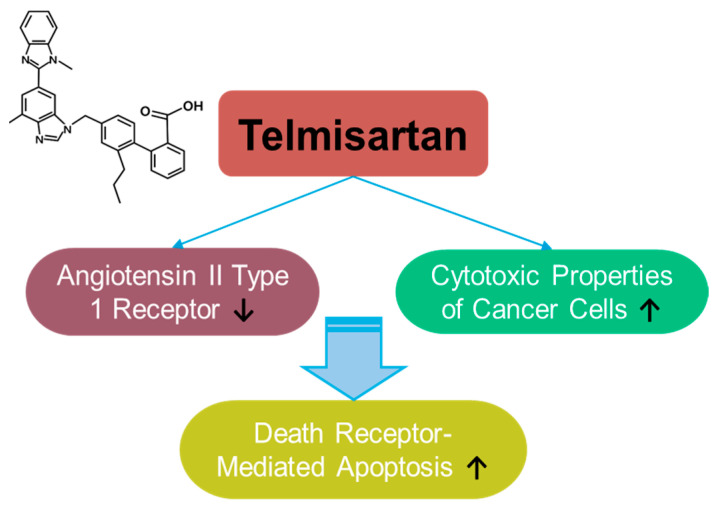
Molecular structure and mechanisms of telmisartan.

Telmisartan is a selective blocker of the angiotensin II type 1 receptor, clinically approved in 1998 (Figure 7). Telmisartan has anti-inflammatory and antioxidant properties, as well as protective roles in hypertension [219,220,221,222] and cognitive impairment [223]. Although the anti-cancer effects of telmisartan have been reported in relatively few articles and have focused on liver, lung, and breast cancers [224,225,226,227,228], its wide usage in treating metabolic syndrome and its application in drug repositioning have been extensively reviewed [229,230,231]. Telmisartan enhances the cytotoxic properties of cancer cells by mediating death-receptor-mediated apoptosis in lung cancer cells [228]. As a delivery strategy, telmisartan was combined with a nanoparticle-mediated programmed drug release platform to permeate the deep breast tumor region [226]. Moreover, the modified structure of the telmisartan–Zn combination has been applied to improve anti-cancer properties through enhanced ROS-mediated cellular apoptosis in lung cancer cells [227]. Although telmisartan is a synthetic compound, the established safety profile of telmisartan possesses an attractive potential in drug repurposing strategies and combinational therapies. However, further investigations are required for the effective application of telmisartan based on the insufficient experimental evidence.

### 3.8. Sulforaphane

**Figure 8:**
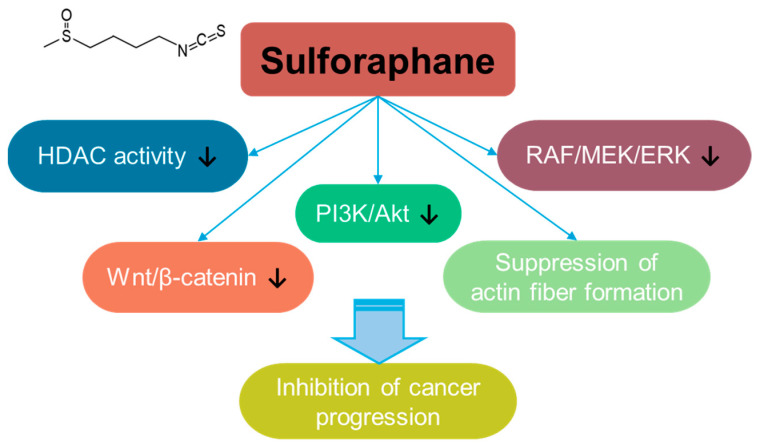
Molecular structure and mechanisms of sulforaphane.

Sulforaphane is a bioactive isothiocyanate phytochemical that is isolated from cruciferous vegetables such as broccoli and kale [232] and extensively reviewed for its multifaceted effects such as its anti-angiogenesis, anti-bacterial, anti-aging, and anti-inflammatory properties [233,234,235]. Sulforaphane treatment suppresses breast cancer metastasis to inhibit actin fiber formation through the inhibition of the RAF/MEK/ERK pathway [236]. In addition, sulforaphane inhibits cancer progression through the inhibition of Wnt/β-catenin or PI3K/Akt signaling in colorectal cancer [237,238]. Sulforaphane also has an inhibitory effect on histone deacetylase (HDAC) activity [239], and its inhibitory effect on HDAC is addressed in breast, prostate, colon, and lung cancer cells [236,237,238,240] (Figure 8). Moreover, the co-administration of sulforaphane with other compounds, such as quercetin, reveals the potential of combinational therapy [52]. Although the anti-cancer roles of sulforaphane are addressed in several preclinical studies, a clinical approach and developmental strategies for sulforaphane should be required to verify its efficacy and safety.

## 4. Conclusions and Perspectives

Understanding cancer-related oxidative mechanisms is an attractive strategy for overcoming cancer and developing effective treatments. In this review, ROS-based anti-cancer drugs were described along with the relevant delivery platforms developed to overcome common issues such as instability, efficacy, short half-life, and unwanted targeting. Over the past decades, advances in delivery systems have significantly enhanced the availability of various potential anti-cancer agents. Although enhancing ROS levels in cancer systems is an effective strategy, targeting cancer cells remains challenging. In addition, there are various hurdles to cancer treatment, such as the recognition of cancer-specific receptors or microenvironments, attenuation of adaptive response, disturbance of the immune system, complications of side effects, and budget limitations. To develop ROS-based cancer treatments, these hurdles must be addressed and verified in various cancers using both in vitro and in vivo experimental systems. Naturally derived anti-cancer compounds possess several beneficial effects, such as long-term use, positive immunomodulatory effects, reduced side effects, and the prevention of drug resistance, compared to other chemical compounds. However, among the compounds mentioned in this review, resveratrol and curcumin have been used in clinical studies against cancer, and telmisartan alone was clinically approved as an anti-hypertensive drug. Thus, this review highlights the challenging issues for basic cancer research on naturally derived anti-cancer compounds (Table 6) and emphasizes the importance of verifying their exact mechanisms of action in cancer systems for developing effective cancer treatment strategies and expanding the range of approved drugs.

## Figures and Tables

**Table 1:** Anti-cancer effects of quercetin.

Cancer Types	Applied Doses (μM)	Effects	Cell Lines and Tissues	Ref.
Breast cancer	5–200	Inhibits invasion, Affects cell cycle regulation, Inhibits cell proliferation with increased DNA damage	MCF-7, MDA-MB-231, SK-BR-3, MDA-MB-453	[31,32,35,43,51]
Prostate cancer	20–80	Inhibits proliferation, Promotes apoptosis	PC-3	[43]
Lung cancer	10–60 In vivo: 50 mg/kg	Inhibits proliferation, Antioxidant role, Anti-inflammatory role	A549, H1299, H1975 In vivo: Subcutaneous tissue	[36,37,43]
Leukemia	20–100	ROS production, Apoptosis induction	U937, Jurkat, HL-60	[53]
Ovarian cancer	25–50 In vivo: 50–60 mg/kg	Inhibits proliferation, Increases chemotherapy sensitivity	A2780S, SKOV3 P#1, CAOV3 In vivo: Subcutaneous tissue	[44,45,46]
Osteosarcoma	10–200 In vivo: 100 mg/kg	Induces apoptosis, Inhibits tumor growth	MG-63, U2OS, 143B In vivo: Subcutaneous tissue	[47,48,49]

Abbreviations include the following: MCF-7, MDA-MB-231, SK-BR-3, MDA-MB-453: human breast cancer cell lines; PC-3, A549, H1299: human lung cancer cell lines; H1975: human non-small cell lung cancer cell line; U937, Jurkat, HL-60: human leukemia cell lines; P#1, CAOV3, A2780S, SKOV3: human ovarian cancer cell lines; MG-63, U2OS, 143B: human osteosarcoma cell lines.

**Table 2:** Anti-cancer effects of curcumin.

Cancer Types	Applied Doses (μM)	Effects	Cell Lines and Tissues	Ref.
Melanoma	2.5–80 In vivo: 20 mg/kg	Apoptosis, Anti-proliferation	B16-F10, A375, G361, SK-MEL-2 In vivo: Subcutaneous tissue	[75,76,77,78]
Liver cancer	5–60	Apoptosis, Anti-metastasis	MHCC97H, HepG2	[79,80,81]
Oral cancer	5–50	Apoptosis, Anti-proliferation	H314, ORL115, OSCC	[77,82]
Cervical cancer	10.9–25	Apoptosis, Anti-proliferation	C33A, CaSki, HeLa, ME180	[83,84]
Colon and colorectal cancer	5–50 In vivo: 50 mg/kg	Apoptosis, Cell cycle arrest	Colo205, HCT116, SW480, RKO, SW48, SW620-Luc2, HT29, LoVo In vivo: colon tissues	[85,86,87]
Lung cancer	5–60	Apoptosis, Anti-proliferation	A549, A549/D16, A549/V16, NCI-H446, NCI-H460	[88,89,90,91,92,93]
Nasopharyngeal carcinoma	1–40	Apoptosis, Anti-proliferation	NPC-TW076	[94]
Thyroid cancer	12–50	Apoptosis, Anti-proliferation	K1PTC	[95]
Gastric cancer	1.25–60 In vivo: 25–50 mg/kg	Apoptosis, Anti-proliferation	BGC-823, SGC-7901, MGC-803 In vivo: tumor tissues	[96,97,98,99]
Osteosarcoma	10 In vivo: 7.5–30 mg/kg	Apoptosis, Anti-proliferation	MG-63, Saos-2 In vivo: tumor tissues	[100,101]
Leukemia	5–21.43 In vivo: 25–200 mg/kg	Apoptosis, Cell cycle arrest	Raji, HL-60, K562, CCL-243 In vivo: tumor tissues	[102,103,104,105]
Bladder cancer	10–20 In vivo: 500 mg/kg	Apoptosis, Anti-proliferation	253J-Bv, T24 In vivo: tumor tissues	[106]
Pancreatic cancer	20	Apoptosis, Cell cycle arrest	Panc-1	[107]
Ovarian cancer	4–16	Apoptosis, Anti-proliferation	A2780	[108]
Breast cancer	11.21–100 In vivo: 12–100 mg/kg	Apoptosis, Anti-proliferation	MCF-7, MDA-MB-231, 4T1 In vivo: tumor tissues	[109,110,111,112,113]
Laryngeal cancer	10	Apoptosis, Anti-proliferation	Hep2	[114]
Head and neck squamous cell carcinoma	0.5	Apoptosis, Anti-proliferation	AMC-HN4	[115]
Glioma	0.5–25	Apoptosis, Anti-proliferation	U87, Glioblastoma stem cells, U251, U235	[116]

Abbreviations include the following: B16-F10: mouse melanoma and fibroblast cell line; A375, G361, SK-MEL-2: human melanoma cell lines; MHCC97H, HepG2: human hepatocellular carcinoma cell lines; H314, ORL115, OSCC: human oral squamous cell carcinoma cell lines; C33A, CaSki, HeLa, ME180: human cervical carcinoma and adenocarcinoma cell lines; Colo205, HCT116, SW480, RKO, SW48, SW620-Luc2, HT29, LoVo: human colorectal carcinoma and adenocarcinoma cell lines; A549/D16, A549/V16, NCI-H460: human lung cancer cell lines (variants and other lung cancer types); NPC-TW076: human nasopharyngeal carcinoma cell line; K1PTC: human papillary thyroid carcinoma cell line; BGC-823, SGC-7901, MGC-803: human gastric carcinoma cell lines; SaOS-2: human osteosarcoma cell line; Raji: human Burkitt’s lymphoma cell line; K562 (also known as CCL-243): human chronic myelogenous leukemia cell line; 253J-Bv, T24: human bladder carcinoma cell lines; Panc-1: human pancreatic adenocarcinoma and carcinoma cell line; glioblastoma stem cells, U87, U251, U235: human glioblastoma cell lines; 4T1: mouse breast cancer cell line; Hep2: human laryngeal carcinoma cell line; AMC-HN4: human head and neck squamous cell carcinoma cell line.

**Table 3:** Various delivery platforms of curcumin for different cancers.

Delivery Platforms	Applied Doses	Effects	Cell Lines and Tissues	Ref.
kappa- carrageenan- mediated delivery	40 μg/mL	Enhances bioavailability and stability	A549	[117]
	6.25 μg/mL (PDT)	Enhances ROS generation and apoptosis	4T1	[118]
Prostate cancer Lung cancer Leukemia	20–80	Inhibits proliferation, Promotes apoptosis	PC-3	[43]
	10 µM	Improves therapeutic efficacy	A549, MCF-7	[111]
	25 µg/mL (PDT)	Enhances cellular uptake, Improves cytotoxicity	MDA-MB-231, MCF-7	[119]
	10 µM	Anti-cancer activity	A549	[120]
	30–130 µM	Induces cytotoxicity, apoptosis, and cell cycle arrest	MCF-7	[121]
	In vivo: 100–500 mg/kg	Reduces tumor growth, Increases apoptosis	In vivo: Subcutaneous tissue	[122]
	30–130 µM	Autophagy induction, Inhibits cell proliferation	A549, NSCLC	[123]
	100 μM,	Enhances cellular uptake	SK-N-AS, SMS-KAN, LA-N-6, IMR-32	[124]
	3.12 μg/mL	Increases solubility and bioavailability	HeLa	[125]
	4 μg/mL	Tumor targeting	HeLa, MCF-7, THP-1	[126]
	3.4 μM (PDT)	Improves cytotoxicity	MKN-45	[127]
	50 μg/mL In vivo: 25 mg/kg	Inhibits tumor growth	4T1, MDA-MB-231 In vivo: Subcutaneous tissue	[128]
	2.74 μM	Enhances therapeutic outcomes	MCF-7	[129]
	25 μg/mL	Improves drug delivery	HepG2, MCTS	[130]
	12 μg/mL In vivo: 1 mg/kg	Anti-tumor effects	MCF-7, breast tissues of female SD rats In vivo: Subcutaneous tissue	[131]
	2.5–12 μg/mL In vivo: 1 mg/kg	Enhances bioavailability	MCF-7, MDA-MB-231, EAC In vivo: Tumor tissue	[132]
	28 μg/mL In vivo: 5 µg/mL	Increases therapeutic efficacy	A549, H1299, H1975, H460, SCC827, PC-9 In vivo: Subcutaneous tumor tissue	[133]
	15 µM	Targeted delivery	U251N	[134]
	5 μg/mL In vivo: 5 mg/kg	Improves bioavailability	C6, MDA-MB-231 In vivo: Zebrafish larvae	[135]
	20 mM	Enhances anti-cancer effects	SK-N-SH	[136]
	5–18 µg/mL	Improves therapeutic efficacy	HepG2	[137]
	20 μM	Anti-cancer activity	U87MG	[138]
	10 mg/mL	Enhances bioavailability	HN5	[139]
	6.25–12.5 μg/mL	Improves drug delivery	HeLa	[140]
	5 mg/mL	Enhances therapeutic outcomes	HN5	[141]
	2.4 μg/mL (PDT)	Increases anti-cancer efficacy	HeLa, T24	[142]
Nanofibrous mat-mediated controlled release	2 mg/mL In vivo: 5–20 mg/kg	Controlled drug release and enhanced stability	PDAC399, T3M4, MIA, PaCa-2, Panc-1 In vivo: Tumor tissue	[143]
Halloysite nanotube-mediated delivery	4–10 μM	Improves bioavailability	HepG2, MCF-7, Caski, HeLa	[144]
	6.5 mg/mL (Ag-TiO, PDT)	Enhances drug delivery	HeLa	[145]
Liposome-mediated delivery	5–50 μM	Improves therapeutic efficacy	AsPC-1, BxPC-3	[146]
	20 µM	Enhances drug delivery	C26	[147]
	20–40 µM In vivo: 25 mg/kg	Enhances radiosensitivity, apoptosis, and cell cycle arrest	C6, U251 In vivo: Subcutaneous tissue	[148]
	150 μg/mL	Increases bioavailability	HeLa	[149]
	32 µg/mL	Improves anti-cancer effects	MCF-7	[150]
Graphene-based nanoformulation delivery	5–20 μg/mL In vivo: 5–20 mg/kg	Improves bioavailability	OC1 In vivo: Guinea pig cochlear tissue	[151]
	1–20 μg/mL	Enhances anti-cancer effects	HuH6, HepT1, HC-AFW1, HepG2	[152]
Active metabolites (tetrahydrocurcumin, hexahydrocurcumin)	50–100 µM	Enhances cytotoxicity and cell cycle arrest	SW480	[153]
	5–25 μM	Inhibits growth, Downregulates COX-2	HT29	[154]
	5–20 mg/kg In vivo: 5–20 mg/kg	Reduces tumor growth, Increases apoptosis	H22 In vivo: Abdominal cavity	[155]
	12.5–50 μM In vivo: 100 mg/kg	Enhances anti-tumor effects	U2OS, MG-63, SaOS-2 In vivo: Lung metastases	[156]
	In vivo: 100–500 mg/kg	Inhibits angiogenesis	In vivo: Tumor tissue	[157]
Photodynamic Therapy (PDT)	2.5–15 μM	Improves therapeutic outcomes	MCF-7	[158]
	5, 7.5 μM	Enhances bioavailability	C6, HUVEC	[159]
	0.39–25 μg/mL	Increases anti-cancer efficacy	HeLa	[160]
	5–100 µM	Improves drug delivery	A549, H1299, MCF-7, MDA-MB-231, NSCC	[161]
	100 µM	Induces ROS production and cell apoptosis	MCF-7, MDA-MB-231, A431, SCC-25, ugMel2	[162]
	67.86 µM	Reduces cell viability, Induces apoptosis and necrosis	MDA-MB-231	[163]
	5–40 μM	Increases bioavailability	A549, THP-1	[164]
	150–200 μM	Improves anti-cancer effects	U87	[165]
	5–25 µM	Enhances therapeutic outcomes	T98G, LN229	[166]
	20 µM	Improves drug delivery	MCF-7	[167]

Abbreviations include the following: PDT: photodynamic therapy; several combinational therapies of both PDT and delivery platforms are represented in the delivery platform section; NSCLC: non-small cell lung cancer cell line; SK-N-AS, SMS-KAN, LA-N-6, IMR-32, SK-N-SH: human neuroblastoma cell lines; U87MG: human glioblastoma cell line (highly invasive and tumorigenic); THP-1: human monocytic leukemia cell line; MKN-45: human gastric carcinoma cell line; MCTS: multi-cellular tumor spheroids (3D tumor model); EAC: Ehrlich ascites carcinoma cells; SCC827, PC-9: human non-small cell lung cancer cell lines; H460: human non-small cell lung cancer (NSCLC) cell line; U251N: variant of U251 glioblastoma cell line; C6: rat glioma cell line; HN5: human head and neck squamous cell carcinoma cell line used in head and neck cancer research; PDAC399, T3M4, MIA PaCa-2: human pancreatic cancer cell lines; SV-HUC-1: human urothelial cell line; AsPC-1, BxPC-3: human pancreatic cancer cell lines; C26: mouse colon carcinoma cell line; OC1: ovarian cancer-related cell line; HuH6, HepT1, HC-AFW1: human liver cancer cell lines; H22: mouse liver cancer cell line; HUVEC: human umbilical vein endothelial cells; HNSCC: head and neck squamous cell carcinoma; A431, SCC-25: human skin carcinoma cell lines; ugMel2: human melanoma cell line; Hs68: human foreskin fibroblast cell line; T98G, LN229: human glioblastoma cell lines; CaSki: human cervical carcinoma and adenocarcinoma cell line; Ag-TiO: silver–titanium dioxide.

**Table 4:** Anti-cancer effects of xanthatin.

Cancer Types	Applied Doses (μM)	Effects	Cell Lines and Tissues	Ref.
Breast cancer	5–40 In vivo: 20 mg/kg	Induces apoptosis, caspase activation, and cell cycle arrest	MCF-7, MDA-MB-231, MDA-MB-415, SK-BR-3, HCC1937 In vivo: Subcutaneous tissue	[170,171,172,173]
Gastric carcinoma	10	Induces apoptosis	MKN-45	[174]
Lung cancer	12.97–50	Induces apoptosis and mitochondrial ROS accumulation, Dysregulates redox balance	A549, H1299, H460, NCI-H520	[175,176,177,178]
Melanoma	10 In vivo: 0.2 mg/kg	Induces apoptosis	A375, B16-F10 In vivo: Tumor tissue	[179]
Colon cancer	10–40 In vivo: 5 mg/kg	Induces apoptosis, caspase activation, and cell cycle arrest	HT29, HCT116, CT26WT In vivo: Subcutaneous tissue	[180,181,182,183]
Hepatocellular carcinoma	1.6–40	Induces apoptosis	HepG2, Bel-7402, SK-Hep-1, SMMC-7721, Huh-7	[184,185,186]
Pancreatic cancer	30	Induces apoptosis	BxPC-3, PANC-1	[187]
Glioma	1–20	Induces apoptosis, Inhibits tumor growth, Triggers ER stress	C6, U251	[188,189]

Abbreviations include the following: MDA-MB-415, HCC1937: human breast cancer cell lines; NCI-H520: human lung cancer cell line; CT26WT: mouse colon carcinoma cell line; Bel-7402: human hepatocellular carcinoma cell line.

**Table 5:** Anti-cancer effects of carvacrol.

Cancer Types	Applied Doses (μM)	Effects	Cell Lines and Tissues	Ref.
Hepatocellular carcinoma	50–400	Induces apoptosis, Modulates ERK1/2 and p38	HepG2	[195]
Glioblastoma	200–1000	Induces apoptosis, Increases ROS production, Inhibits growth, migration, and invasion	DBTRG-05MG, U87	[199,202]
Oral cancer	10–1000	Inhibits proliferation, Induces apoptosis, Reduces migration	OC2, Tca-8113, SCC-25	[200,208]
Osteosarcoma	300–1000	Suppresses viability, Induces apoptosis, Increases ROS production	HOS, U2OS	[201]
Colon and colorectal cancer	50–1000	Inhibits growth, Induces cell cycle arrest, Exerts anti-proliferative effects and selective cytotoxic effects against cancer cells.	HCT116, LoVo	[203,212]
Prostate cancer	1–1000	Inhibits growth, migration, and invasion, Reduces PI3K/Akt, IL-6, Induces apoptosis, ROS production, and cell cycle arrest	PC-3, DU145	[204,205,206,207]
Breast cancer	6.23–1200	Inhibits viability, Induces apoptosis, Synergistic effect with doxorubicin	MCF-7, MDA-MB-231	[209,211,214]
Lung cancer	3.9–500	Inhibits viability, Induces G2/M phase arrest	A549	[210]
Multiple myeloma	10–20	Induces apoptosis, Increases ROS production	U266	[213]
Cervical cancer	9.33–550	Synergistic effect with doxorubicin, Increases ROS production, Induces apoptosis and cell cycle arrest, Promotes autophagy	HeLa	[214,215]

Abbreviations include the following: NCTC2544: human keratinocyte cell line; DBTRG-05MG: human glioblastoma cell line; OC2, Tca-8113: human oral squamous cell carcinoma cell lines; DU145: human prostate cancer cell line; HOS: human osteosarcoma cell line; U266: human multiple myeloma cell line.

**Table 6:** Comparative anti-cancer effects of highlighted compounds.

Compound (Preclinical/ Clinical Stage)	Source	Mechanisms of Action	Key Effects	Target Cancers
Plumbagin (Preclinical)	*Plumbago zeylanica* L.	Increases ROS, Inhibits NF-κB and PI3K/Akt pathway	Cell cycle arrest, apoptosis, invasion inhibition	Breast, lung, prostate, gastric, colorectal, brain, liver, ovarian cancers
Quercetin (Preclinical)	Fruit- and vegetable-derived flavonoid	Increases ROS, Depletes GSH, Activates SIRT1/AMPK	Apoptosis, autophagy, anti-proliferative effects	Breast, lung, prostate, leukemia, ovarian, osteosarcoma, colon, colorectal cancers
Resveratrol (Clinical)	Plant-derived polyphenol	ROS accumulation, Inhibits β-catenin, STAT3, ER stress	Apoptosis, oxidative phosphorylation disruption	Colon, cervical, melanoma, breast, liver, lung, gastric, pancreatic cancers, glioma
Curcumin (Clinical)	*Curcuma longa* (Turmeric)	Enhances ROS generation, Increases calcium, ER/mitochondrial stress	Apoptosis, cell cycle arrest, anti-inflammatory	Melanoma, liver, lung, breast, ovarian, brain, oral, cervical, thyroid, gastric, pancreatic, colon cancers, leukemia
Xanthatin (Preclinical)	*Xanthium strumarium* L.	Accumulates ROS, Mitochondrial dysfunction, Caspase activation	Apoptosis, cell cycle arrest, redox imbalance	Breast, lung, gastric, colon, liver, brain, pancreatic cancers, melanoma
Carvacrol (Preclinical)	*Origanum vulgare* and herbal sources	Increases ROS, Suppresses MAPK/PI3K-Akt pathway, Inhibits TRPM7	Apoptosis, cell cycle arrest, migration inhibition	Liver, prostate, colon, breast, cervical, brain, glioblastoma, oral cancers, multiple myeloma, osteosarcoma
Telmisartan (Clinical approval)	Synthetic compound, Angiotensin II blocker	ROS-mediated apoptosis, Activates death receptor pathways	Cytotoxicity, apoptosis, anti-inflammatory effects	Lung, breast, liver, colon cancers (Preclinical studies in cancers)
Sulforaphane (Preclinical)	Cruciferous vegetables	Inhibits RAF/MEK/ERK pathway, Inhibits Wnt/β-catenin and PI3K/Akt signaling, Inhibits histone deacetylase	Suppresses metastasis, inhibits cancer progression, anti-inflammatory effects	Breast, prostate, colon, lung cancers

## Data Availability

Not applicable.

## Abbreviations

ROS: reactive oxygen species; DNA: deoxyribonucleic acid; GSH: reduced glutathione; GSSG: oxidized glutathione; NRF2: nuclear factor erythroid 2-related factor 2; K-RAS: Kirsten rat sarcoma viral oncogene homolog; NADPH: nicotinamide adenine dinucleotide phosphate.; MMP: matrix metalloproteinase; PI3K: phosphoinositide 3-kinase; PKB (Akt): protein kinase B; SIRT1: sirtuin 1 (NAD+-dependent deacetylase); AMPK: AMP-activated protein kinase; RAF: rapidly accelerated fibrosarcoma; MEK: mitogen-activated protein kinase kinase.
